# Identification and validation of circulating exosomes‐based liquid biopsy for esophageal cancer

**DOI:** 10.1002/cam4.2224

**Published:** 2019-05-17

**Authors:** An Zhao, Liwei Guo, Ji Xu, Lei Zheng, Zhenying Guo, Zhiqiang Ling, Lidong Wang, Weimin Mao

**Affiliations:** ^1^ Institute of Cancer Research Zhejiang Cancer Hospital Hangzhou China; ^2^ Zhejiang University School of Medicine Hangzhou China; ^3^ Department of Surgery Zhejiang Provincial People's Hospital Hangzhou China; ^4^ Department of Pathology Zhejiang Cancer Hospital Hangzhou China; ^5^ Henan Key Laboratory for Esophageal Cancer Research The First Affiliated Hospital of Zhengzhou University Zhengzhou China; ^6^ Department of Thoracic Surgery Zhejiang Cancer Hospital Hangzhou China

**Keywords:** biomarker, diagnostic, esophageal cancer, exosome, liquid biopsy

## Abstract

**Background:**

Early detection of esophageal squamous cell carcinoma (ESCC) recurrence is a key element for follow‐up care and surveillance. The aim of this study is to detect the level of circulating exosomes (CEs) in ESCC patient and clarify its clinical significance.

**Methods:**

In this study, 200 serum samples of ESCC patients were obtained from the Zhejiang Cancer Hospital Biospecimen Repository. Total CEs were purified by selectively capturing epithelial cell adhesion molecule positive exosomes, using magnetic‐bead technique. enzyme‐linked immunosorbent assay (ELISA) was performed to measure the concentration level of CEs. The oncogenic potential of CEs was analyzed in vitro.

**Results:**

Serum concentration of CEs was significantly higher in ESCC patients than in healthy controls (*P* < 0.01). Receiver‐operating characteristic curve analysis demonstrated that CEs concentration could distinguish patients with ESCC from healthy individuals with a sensitivity of 75% and a specificity of 85%. Kaplan‐Meier analysis demonstrated that the increased CEs concentration was associated with poor overall survival (*P* = 0.01) and progression free survival (*P* = 0.03) in ESCC patients. Multivariate cox regression analysis revealed that CEs concentration was an independent prognostic marker for overall survival in ESCC patients (*P* < 0.01). Results from transwell and wound scratching experiments showed that the CEs could promote cell migration and invasion.

**Conclusions:**

This study clearly demonstrates that CEs from ESCC patients are stable enough to be measured and their levels in ESCC patients are significantly upregulated. Circulating exosomes could serve as a novel noninvasive biomarker for detection of ESCC. Their involvement in carcinogenesis must be further established.

## 1 BACKGROUND

Esophageal cancer (ECa) is the sixth most common cause of cancer‐related mortality worldwide.^1^, ^2^ More than two‐thirds of patients with ECa develop local recurrence or distant metastases and even die despite complete resection of the primary tumor and multimodal treatments.^3^ Early detection of ECa recurrence is a key element for follow‐up care and surveillance.^4^, ^5^ However, because early detection is difficult to achieve by conventional endoscopy or radiological examination, a novel noninvasive and convenient method is urgently needed.

The circulating exosomes (CEs) have attracted increasing interests in the liquid biopsy field due to the potential that CEs may serve as a biomarker for human cancer screening and monitoring.^6^, ^7^ Exosomes are 40‐150 nm small vesicles in blood and other bodily fluids that can be released by tumor cells during tumor progression and metastasis.^8^, ^9^ The selective nucleic acids or protein cargo in exosomes could play a role in cell‐to‐cell communications.^10^, ^11^

The detection of circulating tumor cells (CTCs) has been reported to increase the risk for ECa recurrence after resection, but the CTCs cut‐off value still remain controversial because the number of CTCs is extremely rare.^3^, ^12^, ^13^ Compared with CTCs, CEs exist in large quantities in the bodily fluid, and carry tumor markers of their parent tumor cells. Although the secretory mechanisms and functions of CEs are still unclear, the use of CEs as a potential biomarker may be promising.

Over half of the global ECa cases occur in China with 477.9 thousand new cases diagnosed in 2015.^14^ And esophageal squamous cell carcinoma (ESCC) is the major pathologic subtype of ECa in non‐Western populations (eg, 90% of Chinese patients).^1^ Therefore, the key aim of this study is to determine whether CEs can serve as a novel noninvasive biomarker for the diagnosis and prognosis of patients with ESCC.

## 2 METHODS

### 2.1 Patients and samples

All the 100 patients with histopathologically confirmed ESCC were derived from the Zhejiang Cancer Hospital. All the patients did not receive any preoperative chemoradiotherapy before operation and were followed up from operation time to April 30, 2017. Of the patients, there were 88 males (88%) with an average age of 62.53 ± 7.68 (range 47‐83) years and 12 females with an average age of 61.33 ± 8.049 (range 48‐73) years. Of the healthy volunteers, there were 65 males (65%) with an average age of 47.74 ± 13.33 (range 23‐80) years and 35 females with an average age of 50.34 ± 13.14 (range 24‐74) years. The blood samples were centrifuged at 3,500 g for 10 minutes to extract the serum, and then the serum was stored at Zhejiang Cancer Hospital Biospecimen Repository until analysis. The study was approved by the hospital ethics committee and the informed consent was obtained from all participants.

### 2.2 Isolation and purification of exosomes from serum

Exosomes were isolated from serum using the Total Exosome Isolation kit (Invitrogen). 1 mL serum was mixed with 200 µL of the Total Exosome Isolation reagent and incubated at 4°C for 30 minutes, and then the sample was centrifuged at 12,000 *g* for 20 minutes at room temperature. Exosomes were contained in the pellet at the bottom of the tube. The pellet was suspended in 500 µL of Phosphate Buffered Saline (PBS). Epithelial cell adhesion molecule (EpCAM) positive exosomes were isolated using EpCAM isolation beads (Invitrogen). 20 µL solutions with beads were added for each reaction. After end‐over‐end mixing overnight at 4°C, the beads‐bound exosomes were isolated on a magnetic separator and were then resuspended in 200 µL of PBS for further use.

### 2.3 Western blot and immunofluorescence analysis

Western blot analysis was performed to analyze the expression of exosomal marker proteins including the CD9 and HSP90 (Proteintech). Proteins were separated by SDS‐PAGE and transferred onto polyvinylidene fluoride membranes (Millipore). After membranes were blocked with 5% milk for 30 minutes, they were probed with various primary antibodies overnight at 4°C, followed by incubation with secondary antibodies for 1 hours at room temperature, and finally visualized with enhanced chemiluminescence reagent (Thermo Scientific). Immunofluorescence was performed following the standard protocol recommended by Cell Signaling. Briefly, after washing with PBS twice, samples were blocked with 5% normal goat serum in PBS for 1 hours before incubation with primary antibody cocktail overnight at 4°C, then washed and incubated with secondary antibodies followed by examination using confocal microscope.

### 2.4 Zetasizer Nano ZS analysis

Exosome size was measured with a Zetasizer Nano ZS (Malvern Instruments), which was equipped with a 640‐nm laser. All measurements were performed at room temperature.

### 2.5 Enzyme‐linked immunosorbent assay

Exosomes isolated from serum were adsorbed onto ELISA plates (Thermo Scientific) and plates were blocked overnight in PBS containing 1% bovine serum albumin (BSA, Sigma). After being washed, the plates were incubated with CD9‐antibodies (Proteintech) diluted in blocking solution (1 μg/mL) for 1 hours at room temperature, followed by horseradish peroxidase conjugated secondary antibody (Sigma) incubation, and then the plates were detected at 450 nm to observe the absorbance. Each sample was run in duplicates for analysis.

### 2.6 Stability evaluation of CEs

CEs samples were divided into five aliquots for delayed processing (0, 3, 5, 7 and 10 days) and were divided into three aliquots for experiment at different temperature (−20°C, −80°C and RT). 250 ng of exogenous protein (Recombinant human CD9 protein, Proteintech) was added to a randomly selected sample and incubated for 1 hours at RT. The experiment samples were then measured to evaluate the effect of different conditions on CEs. Each sample was run in duplicates for analysis.

### 2.7 Cell proliferation assay

Esophageal squamous cell carcinoma cells (KYSE‐150 cells and KYSE‐450) were washed with PBS and suspended at 3 × 10^3^ cells/mL. The cells were cultured in RPMI 1640 medium containing 1% fetal bovine serum with or without exosomes (100 µg/mL) isolated from patients with ESCC in triplicate using 96‐well plates. The number of viable cells was assayed using the cholecystokinin‐8 test according to the manufacturer's protocol. Proliferation rates were determined at 0, 3, 6, 9, 12 and 24 hours. Cell viability was determined by reading the absorbance at 450 nm.

### 2.8 Colony formation assay

In this study, 500 cells were seeded into six‐well plates and incubated at 37°C with 5% CO_2_ for 10 days. Culture plates were performed in duplicates. Formed colonies were fixed with 4% paraformaldehyde and stained with crystal violet (Beyotime) for 15 minutes. Colonies containing more than 50 cells were counted.

### 2.9 Wound scratch assay

Cells were cultured in a six‐well flat‐bottom plate and then wounded by scratching with a pipette tip. Floating cells were removed and each well was added medium with or without exosomes (100 µg/mL). The cells were observed and photographed under an inverted microscope at 0 and 24 hours after scratching. Cell motility was analyzed by comparing the gap distance between the two time points.

### 2.10 Transwell assay

Diluted matrigel (30 µL) was placed into the upper chamber of the transwell plate (Corning) and then incubated for 1 hours at 37°C. The cells (1 × 10^5^) were subsequently plated in the upper chamber of the transwell plate and cultured in medium with or without exosomes. Here, 500 µL of medium containing 10% fetal bovine serum was added into the lower chamber. After 48 hours, the cells were fixed with 4% paraformaldehyde and stained with crystal violet (Beyotim). A microscope was used to image and count the migrated cells.

### 2.11 Statistical analysis

All statistical analyses were conducted using SPSS 20.0 statistical software and Graphpad Prism 5.0 software. Concentration difference was analyzed by using the Mann‐Whitney U test, Friedman or Wilcoxon test. The cut off values of CEs concentration were determined by receiver‐operating characteristics (ROC) curve analyses. The overall survival (OS) and progression free survival (PFS) curves were generated by Kaplan‐Meier method. The correlation between CEs and survival was calculated through Cox proportional hazards regression model. The significance of the multiple comparisons carried out in this study was considered statistically significant when *P* value was less than 0.05.

## 3 RESULTS

### 3.1 Patient information and CEs characteristics

A total of 100 patients were enrolled into this study and their clinicopathologic features were summarized in Table 1. The median age of the patients was 62 years, with a range of 47‐83 years. Among the 100 patients, 11 cases had a well‐differentiated tumor, 65 moderately differentiated and 24 poorly differentiated. According to the 7th Union for International Cancer Control (UICC) staging system, 25 patients were stage 1, 49 patients were stage 2, 13 patients were stage 3 and 13 patients were stage 4.

**Table 1:** Patients characteristics and correlation between clinical characteristics and CEs concentration

	No. Pts	CEs concentration	
		Mean ± SD	*P* value^∗^
Gender			
Male	88	2.962 ± 0.773	0.881
Female	12	2.997 ± 0.678	
Age			
≥62 y	52	2.850 ± 0.674	0.113
<62 y	48	3.091 ± 0.831	
Tumor size			
pT1 + pT2	51	2.889 ± 0.763	0.301
pT3 + pT4	49	3.046 ± 0.754	
Lymph node invasion			
Negative	47	2.918 ± 0.684	0.552
Positive	53	3.009 ± 0.825	
Metastasis			
Negative	96	2.960 ± 0.770	0.744
Positive	4	3.088 ± 0.461	
Tumor grade			
1	11	2.944 ± 0.581	0.776
2	65	3.003 ± 0.807	
3/4	24	2.875 ± 0.713	
UICC stage			
Stage 1	25	2.798 ± 0.798	0.570
Stage 2	49	3.062 ± 0.810	
Stage 3	13	2.930 ± 0.510	
Stage 4	13	2.963 ± 0.710	

Abbreviations: CEs, circulating exosomes; UICC, Union for International Cancer Control.

^∗^ Indicates significance according to Mann‐Whitney test.

**Figure 1:**
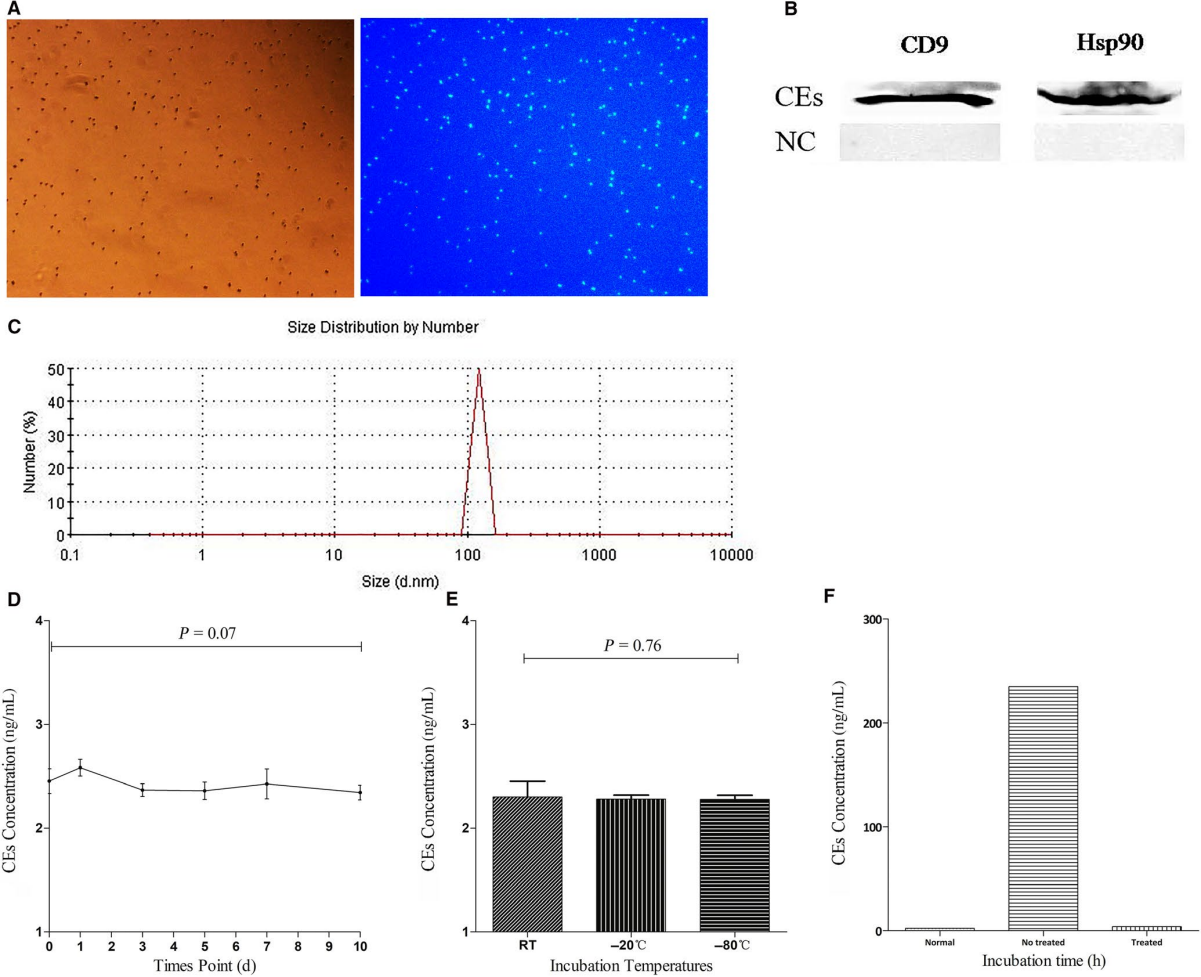
Visualization and identification of isolated circulating exosomes (CEs). (A) Immunostaining of the beads clearly showed exosomal marker CD9 on the surface of the CEs‐beads complexes. (B) Western blot analysis also demonstrated the presence of exosomal protein marker CD9 and Hsp90 in CEs. (C) Zetasizer Nano ZS analysis results revealed that the CEs were round in shape with an average size of 100 nm. (D) CEs concentration as determined by ELISA was plotted on the Y axis. The incubation time of serum (days) was plotted on the X axis. No significant differences in the CEs concentrations with delayed processing. (E) No differences were observed among the CEs incubated at −80°C freezer, −20°C freezer or kept at RT. (F) The concentration of CEs after addition of exogenous exosomal protein (Treated Group) and without any treatment revealed an obvious decrease in exogenous‐free protein over time

To obtain CEs, total exosomes were incubated with anti‐EpCAM magnetic beads and the CEs were purified by the magnetic separation system. CEs were then visualized following immunostaining with exosomal marker proteins (Figure 1A). Western blot analysis also demonstrated the presence of exosomal marker proteins CD9 and Hsp90 in CEs (Figure 1B). Zetasizer Nano ZS analysis results revealed that the CEs were round in shape with an average size of 100 nm (Figure 1C).

No significant differences were observed in CEs concentration under different exposure times and incubation temperatures (Figure 1D,E). The concentration of CEs after addition of exogenous protein revealed an obvious decrease in exogenous‐free protein over time, suggesting that the magnetic beads could reduce the interference of exogenous free protein or that the bilayer lipid membrane of CEs could resist the enzyme (Figure 1F).

### 3.2 Detection and diagnostic value of CEs in ESCC

**Figure 2:**
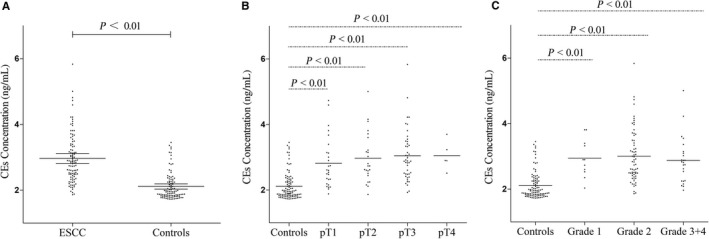
Concentration levels of CEs in the serum from esophageal squamous cell carcinoma (ESCC) patients and healthy controls. (A) Scatter plots of CEs concentration in serum from patients with ESCC (n = 100) and healthy controls (n = 100). Mean CEs concentration was significantly higher in the serum from patients with ESCC (Mean: 2.97 ng/mL, 95% CI: 2.82‐3.12) compared with circulating exosomes (CEs) concentration from control subjects (Mean: 2.11 ng/mL, 95% CI: 2.03‐2.20) (*P* < 0.01). Scatter plots of concentrations levels of CEs in the ESCC patients with different tumor sizes (B) and grades (C). Each of these parameters including pT1 (*P* < 0.01) and grade 1 (*P* < 0.01) patients had significantly higher CEs concentration compared with the control subjects. The lines represented the medians. Mann‐Whitney U test was used to determine statistical significance

The average concentration of CEs was significantly higher in the serum from patients with ESCC (mean: 2.97 ng/mL, 95% CI: 2.82‐3.12) compared with CEs concentration from control subjects (mean: 2.11 ng/mL, 95% CI: 2.03‐2.20) (*P* < 0.01; Figure 2A). We examined the correlation between the CEs concentration and clinical parameters. No significant association was observed between CEs concentration and gender, age, tumor size, lymph node invasion, metastasis, tumor grade, and UICC stage (Table 1). However, each of these parameters including pT1 and grade 1 patients had significantly higher CEs concentration when compared with the control subjects (*P* < 0.01 and *P* < 0.01, respectively; Figure 2B,C).

**Figure 3:**
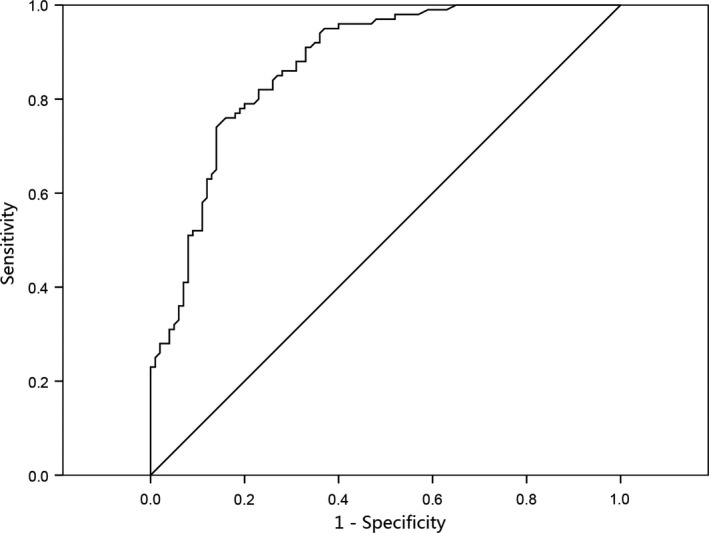
Receiver operating characteristic (ROC) curve analysis using circulating exosomes (CEs) for discriminating (esophageal squamous cell carcinoma) ESCC patients. CEs yielded an AUC (the areas under the ROC curve) of 0.87 (95% CI = 0.82‐0.92) with a sensitivity of 75% and a specificity of 85% in discrimination between ESCC patients and the healthy controls

To assess the feasibility of using CEs as a diagnostic tool for the detection of ESCC, ROC curve analysis was performed. The ROC analysis demonstrated an area under the ROC curve (AUC) of 0.87 (95% CI: 0.82‐0.92) (Figure 3). When the cut‐off value of CEs concentration was set at 2.43, the sensitivity was 75% and the specificity was 85%.

### 3.3 Correlation between CEs and prognosis of ESCC

**Figure 4:**
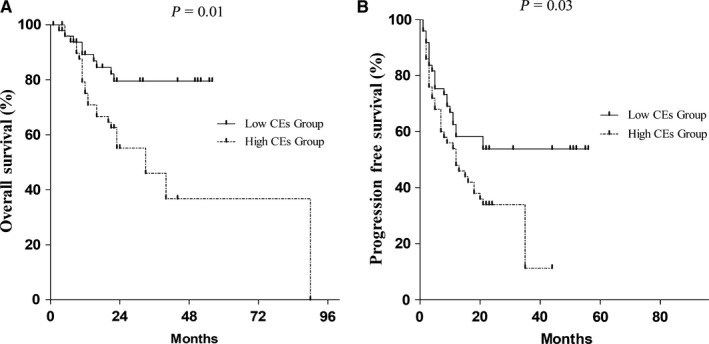
Outcomes of esophageal squamous cell carcinoma (ESCC) patients with high CEs concentrations compared with low circulating exosomes (CEs) concentrations. Kaplan‐Meier survival curves of ESCC patients with low and high CEs concentration. High concentration of CEs was associated with a shorter OS (*P* = 0.01) and PFS (*P* = 0.03). The P value was calculated using the log‐rank test

The median follow‐up time of surviving patients was 22 months. The cases above the median level of CEs concentration were found to have high CEs concentration and the cases below the median level were showed to have low CEs concentration. Patients with high CEs concentration had significantly worse OS (*P* = 0.01) (Figure 4A) and PFS (*P* = 0.03) (Figure 4B) compared with patients with low CEs concentration.

The multivariate analysis was used to test the effects of CEs on OS and PFS independent from other risk factors including age, gender, tumor size, lymph node invasion, tumor grade, and tumor stage. The risk of tumor recurrence in high CEs concentration group was 4.54 times higher than in the low concentration group (HR: 4.54, 95% CI: 1.78‐11.58, *P* < 0.01). In addition, the CEs concentrations were an independent prognostic marker for OS (*P* < 0.01) (Table 2).

**Table 2:** Multivariate analyses of overall survival and progression‐free survival of ESCC patients in this study

Variables	Overall survival		Progression‐free survival	
	HR (95% CI)	*P* value^∗^	HR (95% CI)	*P* value^∗^
Age				
≤61 y vs ≥62 y	1.96 (0.89‐4.32)	0.10	0.98 (0.55‐1.74)	0.93
Gender				
Male vs female	1.28 (0.37‐4.37)	0.70	1.42 (0.65‐3.10)	0.38
CEs concentration				
High vs low	4.54 (1.78‐11.58)	0.00	8.97E6 (0‐1.55E7)	0.86
Tumor size				
pT1 + pT2 vs pT3 + pT4	1.52 (0.64‐3.62)	0.35	0.63 (0.33‐1.20)	0.16
Lymph node invasion				
Positive vs negative	2.18 (0.85‐5.60)	0.11	1.87 (0.99‐3.53)	0.05
Tumor grade				
G1 + G2 vs G3 + G4	3.56E5 (0‐)	0.97	1.01 (0.41‐2.51)	0.98
UICC stage				
T1 + T2 vs T3 + T4	3.05 (1.30‐7.18)	0.01	0.84 (0.39‐1.81)	0.65

Abbreviations: CEs, circulating exosomes; ESCC, esophageal squamous cell carcinoma; HR, hazard ratio.

^∗^ Indicates significance according to Cox regression analysis comparing the specified variables.

### 3.4 In vitro* analysis of the oncogenic potential of CEs*

**Figure 5:**
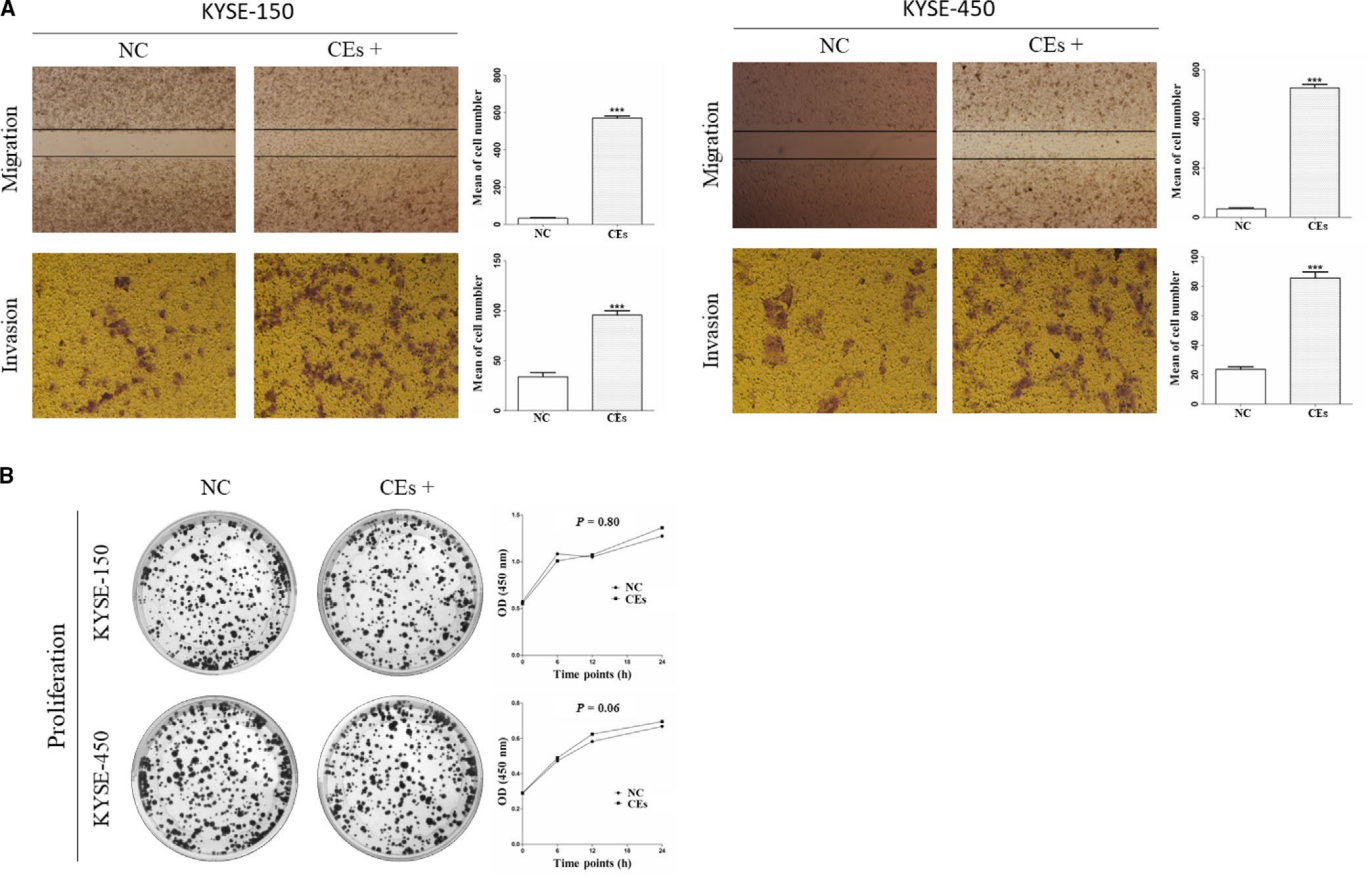
Effect of CEs on ESCC cell. (A) CEs increased cellular motility of KYSE‐150 and KYSE‐450 cells. Representative pictures (left) and quantitative data (right) of transwell (migration or invasion) assays. (B) Clone formation assay (left) showed that there were no differences between cells cultured with or without exosomes. The proliferation assay by CCK‐8 assay showed that there was no significant difference between cells treated with or without CEs. All experiments were performed at least three times and data were statistically analyzed by two‐sided t‐test or friedman test. ^***^ *P* < 0.01 versus control. Error bars indicated SEM

To assess the oncogenic potential of CEs, ESCC cells lines (KYSE‐150, KYSE‐450) were treated with or without CEs. In the wound scratching assay, it was observed that CEs increased cellular motility of ESCC cells. At the time point of 24 hours, CEs promoted more cells to migrate across the wound edge into the scratch area (Figure 5A). When studied in a transwell migration assay, cells treated with CEs also demonstrated increased invasion compared with control cells (Figure 5A). However, the cell proliferation assay showed that there were no significant differences between cells treated with or without CEs (Figure 5B).

## 4 DISCUSSION

Despite the availability of conventional diagnostic tools, such as the imaging test and endoscopy, pretreatment staging remains inaccurate. Therefore, a novel tool for early detection and accurate therapy monitoring in ESCC is urgently needed. As far as we know, this is the first study reporting the diagnostic significance of CEs in ESCC patients, which indicated that CEs could be a promising liquid biopsy for diagnosis of ESCC.

To confirm that the CEs could play a useful role as a biomarker for diagnosis, CEs were treated with different conditions include prolonged storage and different incubation temperatures, and the results showed that CEs were stable enough to be measured under different storage conditions. CEs concentrations after addition of exogenous exosomal protein revealed an obvious decrease in exogenous free protein over time. These results suggested that the bilayer lipid membrane or nanostructure of CEs could resist the enzyme and physical modification.

All the obtained CEs were of good quality and suitable for detection. CEs concentrations were significantly higher in ESCC patients than in healthy controls. The ROC analysis showed that CEs could distinguish the ESCC patients from the healthy controls. CEs yielded an AUC of 0.87 (95% CI: 0.82‐0.92) with a sensitivity of 75% and a specificity of 85% in discrimination between the ESCC patients and the controls. The OS and PFS were significantly shorter in the presence of high CEs.

We examined the correlation between the CEs concentrations and the clinical parameters. No significant association was found between the CEs and clinical parameters (Table 1). We next compared the CEs of control subjects with that of ESCC patients with different tumor sizes and grades. Each of these parameters including pT1 and grade 1 patients had significantly higher CEs concentration when compared with the control subjects (*P* < 0.01 and *P* < 0.01, respectively; Figure 2B,C), which suggested that CEs test could find ESCC early.

Exosomes could be released by healthy and abnormal cells.^15^, ^16^ Serval studies have been reported that EpCAM contributes to ESCC cell proliferation and tumorigenesis and may be a useful therapeutic target for ESCC.^17^, ^18^ EpCAM magnetic beads were used in our study for tumor‐associated exosome purification to improve its accuracy and specificity for epithelial cancerous origin and avoid secondary interference from nonepithelial origin in a certain extent.

We further confirmed that ESCC cells treated with CEs from patients had higher invasion speed than ESCC cells without CEs treatment by invasion and transwell migration assay. The short noncoding RNAs carried by exosomes have attracted increasing interests in the intercellular communication field based on the premise that exosome may act as the effector of tumorigenesis and also increase the complexity of tumor processes.^19^, ^20^ Several studies have been demonstrated that exosomes secreted by tumor could shuttle short RNA to cells in the tumor microenvironment to promote tumor metastasis and inhibit the immune response.^21^, ^22^ The identification of the functional RNA, noncoding RNA or other nucleic acid within CEs by high throughput sequencing technologies needs to be carried out in the future.

According to the “Reporting Recommendations for Tumor Marker Prognostic Studies,” some limitations of this study deserve more discussion.^23^ The sample size of this study was relatively small, thus the future evolutions in larger patient cohorts would be necessary to validate whether CEs could be used as an early marker for treatment response or it could help to distinguish high‐risk patients after surgery or after radio‐chemotherapy from low risk patients. Although ESCC is the most common subtype of ECa in China, it needs to test the CEs concentration in other subtypes of esophageal cancers and nonmalignant esophageal disease such as inflammatory diseases. However, our study clearly demonstrates that CEs from ESCC patients are stable enough to be measured, and might serve as a novel biomarker for noninvasive ESCC diagnosis and prognosis in the future.

## 5 CONCLUSIONS

CEs might emerge as a potential biomarker for diagnostic and monitoring purposes in ESCC patients in the future.

## CONFLICT OF INTEREST

The authors declare that they have no conflict of interest.

## AUTHORS’ CONTRIBUTIONS

An Zhao Conceptualization, methodology, validation, formal analysis, investigation, data curation, writing‐original draft, writing‐review, project administration, and funding acquisition. Liwei Guo: Data acquisition, quality control of data, statistical analysis, writing‐original draft and edition. Ji Xu: Investigation, data acquisition and software. Lei Zheng: Methodology, data acquisition, quality control of data. Zhenying Guo: Methodology and quality control of data. Zhiqiang Ling: Conceptualization and methodology. Lidong Wang: Conceptualization, supervision, writing‐original draft, writing‐review and editing. Weimin Mao: Conceptualization, validation, investigation, writing‐original draft, writing‐review, visualization, supervision, project administration, and funding acquisition.

## ETHICS APPROVAL AND CONSENT TO PARTICIPATE

This study was approved by the Ethics Committee of Zhejiang Cancer Hospital (Registration Number: 2015‐02‐125).

## Data Availability

The datasets supporting the conclusions of this article are included within the article.
